# Facile Preparation of Monodisperse Cu@Ag Core–Shell Nanoparticles for Conductive Ink in Printing Electronics

**DOI:** 10.3390/mi14071318

**Published:** 2023-06-27

**Authors:** Gang Li, Xuecheng Yu, Ruoyu Zhang, Qionglin Ouyang, Rong Sun, Liqiang Cao, Pengli Zhu

**Affiliations:** 1 System Packaging and Integration Research Center, Institute of Microelectronics of Chinese Academy of Sciences, Beijing 100029, China; gang.li@siat.ac.cn; 2 Shenzhen Institute of Advanced Electronic Materials, Shenzhen Institute of Advanced Technology, Chinese Academy of Sciences, Shenzhen 518055, China; 3 University of Chinese Academy of Sciences, Beijing 100049, China

**Keywords:** core–shell nanoparticles, antioxidation, conductive ink, printing electronics

## Abstract

Copper-based nanoinks are emerging as promising low-cost alternatives to widely used silver nanoinks in electronic printing. However, the spontaneous oxidation of copper under ambient conditions poses significant challenges to its broader application. To address this issue, this paper presents an economical, large-scale, and environmentally friendly method for fabricating Cu@Ag nanoparticles (Cu@Ag NPs). The as-prepared nanoparticles exhibit a narrow size distribution of approximately 100 nm and can withstand ambient exposure for at least 60 days without significant oxidation. The Cu@Ag-based ink, with a 60 wt% loading, was screen-printed onto a flexible polyimide substrate and subsequently heat-treated at 290 °C for 15 minutes under a nitrogen atmosphere. The sintered pattern displayed a low electrical resistivity of 25.5 μΩ·cm (approximately 15 times the resistivity of bulk copper) along with excellent reliability and mechanical fatigue strength. The innovative Cu@Ag NPs fabrication method holds considerable potential for advancing large-scale applications of copper-based inks in flexible electronics.

## 1. Introduction

Over the past several decades, there has been growing interest in the development of printed electronics due to their potential applications in emerging technologies such as transparent conductive circuits, flexible wearable devices, light-emitting diodes (LEDs) , radio frequency identification tags, electromagnetic interference (EMI) shielding [1,2,3,4,5], and chip packaging [6,7]. Silver (Ag) nanomaterials dominate the conductive ink field owing to their high electrical conductivity and inherent inertness. However, the high cost of Ag nanomaterials presents an obstacle to their application and has spurred the development of more affordable conductive ink materials. Copper (Cu) is considered the most promising alternative to silver, offering a 99% cost reduction with only a 6% loss of conductivity [8,9,10]. Nevertheless, copper’s susceptibility to oxidation can lead to the formation of a thermodynamically stable surface oxide layer, causing significant performance deterioration, such as reduced electrical conductivity and increased thermal annealing temperatures [10,11,12].

Various methods have been proposed to address this issue, falling into two main categories. The first category involves using Cu precursor-based metal–organic decomposition (MOD) ink, primarily consisting of copper organic compounds and CuO, which are reduced to zero-valent copper during subsequent ink deposition or sintering processes [13,14,15,16]. Sintering techniques employed in metal precursor inks often involve plasma [17,18], selective lasers [19,20], and intense pulsed lighting (IPL) [21,22,23,24], necessitating sophisticated equipment. A notable drawback of this approach is its temporary antioxidation effect; the bare Cu transformed from the copper precursor remains vulnerable to O₂ and H₂O attacks in the ambient atmosphere. The second approach involves constructing a core–shell structure that encapsulates the unstable Cu core with an inert shell. Typically, materials used to protect the Cu core include insulating materials such as polyvinylpyrrolidone (PVP), lactic acid, SiO₂, Al-doped ZnO, and graphite oxide, as well as highly conductive carbon materials and metals (Ag, Au, Ni, Sn) [10,25,26]. Considering the cost and electrical conductivity, silver can be regarded as an ideal protective shell material for the Cu core.

Michael et al. proposed a selective reduction route for synthesizing Cu@AgNPs, wherein the Ag ion reduction process occurs only on the Cu core particle surfaces, initiated by a galvanic displacement reaction between Cu and Ag ions [27]. The resulting Cu@Ag NPs, with tunable silver shell thicknesses, demonstrated high oxidation resistance up to 200 °C when the silver shell remained intact. Sang-soo et al. synthesized ultrafine Cu@Ag particles less than 20 nm in size using a similar principle and found that the oxidation behavior of the Cu@Ag NPs was closely related to the quality of the Ag shell [28]. However, these methods raised environmental and cost concerns due to Cu sacrificial consumption and the involvement of numerous tedious steps, such as ultrasonication, centrifugation, desiccation, and ball grinding, in the two separate reduction processes.

To overcome these drawbacks while retaining the advantages of electroless plating methods, we demonstrated a facile pseudo-one-pot route for synthesizing Cu@Ag NPs. This low-cost, large-scale, and environmentally friendly method produces monodisperse Cu@Ag NPs without the presence of undesirable individual silver particles. The enhanced antioxidation properties of the Cu@Ag NPs were confirmed through various analytical techniques. The Cu@Ag NPs were dispersed in an organic solvent and screen-printed onto a polyimide (PI) substrate. The excellent properties of the sintered pattern, such as conductivity, reliability, and mechanical durability, make the copper-based ink suitable for flexible and wearable device fabrication.

## 2. Materials and Methods

### 2.1. Material

Copper hydroxide (Cu(OH)₂), polyvinylpyrrolidone-K30 (PVP-K30), L-ascorbic acid, silver nitrate (AgNO₃), and hydroxyethyl cellulose were obtained from Aladdin Reagent Co., Ltd. (Shanghai, China) Ethanol, ethylene glycol, diethylene glycol, and glycerol were purchased from the Sinopharm Chemical Reagent Co. Ltd. (Shanghai, China). All chemicals were used as received without further purification. The ultrapure water (18 MΩ cm⁻¹) utilized in all experiments was produced by a Milli-Q system.

### 2.2. Preparation of Cu@Ag Nanoparticles and Conductive Ink

A pseudo-one-pot route was employed for the synthesis of Cu@Ag nanoparticles. In a round-bottom flask, 0.98 g of Cu(OH)₂, 6 g of L-ascorbic acid, and 2 g of PVP-K30 were added to 150 mL of ethanol, stirred at room temperature, and then heated to 80 °C. The reaction mixture was maintained at 80 °C for 15 minutes until the solution’s color changed from blue to reddish, indicating the formation of Cu nanoparticles. Subsequently, 0.34 g of AgNO₃ dispersed in 10 mL of water was added dropwise to the flask. Upon adding AgNO₃, the mixture’s color changed from reddish to dark grey. Finally, the resulting mixture underwent six centrifugation and redispersion cycles in ethanol and water, respectively, to obtain the Cu@Ag nanoparticles. The Cu@Ag nanoparticles, with traces of hydroxyethyl cellulose, were dispersed in a solution of glycerol, ethylene glycol, and diethylene glycol. The insulating patterns screen-printed on the PI substrate using the Cu@Ag-based ink were converted to conductive patterns using heat sintering treatment.

### 2.3. Characterization

The particle morphology and size were observed using a field-emission scanning electron microscope (FE-SEM, FEI Nova Nano SEM 450). Low-resolution and high-resolution transmission electron microscope (TEM) images and energy-dispersive spectroscopy (EDS) were obtained using an FEI Tecnai G2F20S-TWIN microscope equipped with HAADF and EDS detectors. UV-vis absorption spectra were recorded on a UV-Vis-NIR spectrometer (Shimadzu UV-3600, Kyoto, Japan). Crystal structures were determined using powder X-ray diffraction (XRD) patterns acquired with an X-ray diffractometer (Rigaku D/Max 2500, Tokyo, Japan) with monochromated Cu-Kα radiation (λ = 1.541874 Å) in the range of 20° to 80° at a scanning rate of 10°/min. The thermal behavior of the particles was recorded using a thermogravimetric analyzer (TA Q600, Hüllhorst, Germany). Fourier-transform infrared (FT-IR) spectra were recorded with an FT-IR spectrophotometer (Vertex70FT-IR-Spektrometer, saarbrücken, Germany) in the range 4000–1000 cm⁻¹. Surface oxidation characteristics of the particles were examined using X-ray photoelectron spectroscopy (XPS) spectra recorded on a PHI-5702 multifunctional spectrometer with an Al Kα X-ray source. The rheological properties of the conductive ink were measured using an Anton Paar MCR302 rheometer. The electrical resistivity of the converted pattern was measured with an Agilent Digital Multimeter using the following equation:

$$\rho =\frac{Rah}{l}$$

where R, a, h and l denote the displayed resistance, line width, thickness of the film, and line length, respectively [29].

## 3. Results and Discussion

**Figure 1:**
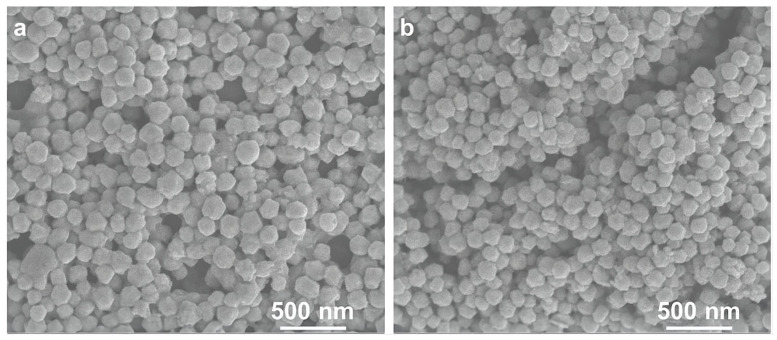
SEM images of the as-prepared Cu nanoparticles (**a**) and Cu@Ag nanoparticles (**b**).

The one-pot route to prepare the Cu@Ag nanoparticles was based on the successive reduction of the two metals by the same reductant in a seeded growth fashion. First, the slightly soluble copper precursor Cu(OH)₂ was reduced by L-ascorbic acid via a mild reduction process. The mechanism of the nucleation and growth of the copper nanoparticles was related to the dissolve–ionize–reduce equilibrium of the copper hydroxide, which has been elaborated in our previous work [30]. The uniform morphology and excellent monodispersity of the copper particles (Figure 1a) were attributed to the homogeneous nucleation based on Lamer’s model [28]. The size distribution of Cu@Ag nanoparticles is 100 nm according to DLS (Figure S2). After the reduction of copper hydroxide was completed, the Ag ions were added dropwise into the growth solution and immediately reduced to Ag atoms by the remaining L-ascorbic acid. In this feeding mode, the concentration of newly generated silver atoms could be kept relatively low. From a thermodynamic standpoint, the increase in surface Gibbs free energy for metal reduction onto an already formed particle is significantly lower than that of individual nucleation in solution. These factors can explain the formation of well-defined Cu@Ag nanoparticles without free Ag nanoparticles (Figure 1b). Furthermore, compared with the successive two-step reduction route, this method was significantly simplified as tedious processes such as washing, centrifugation, drying, and ball-milling were not required.

**Figure 2:**
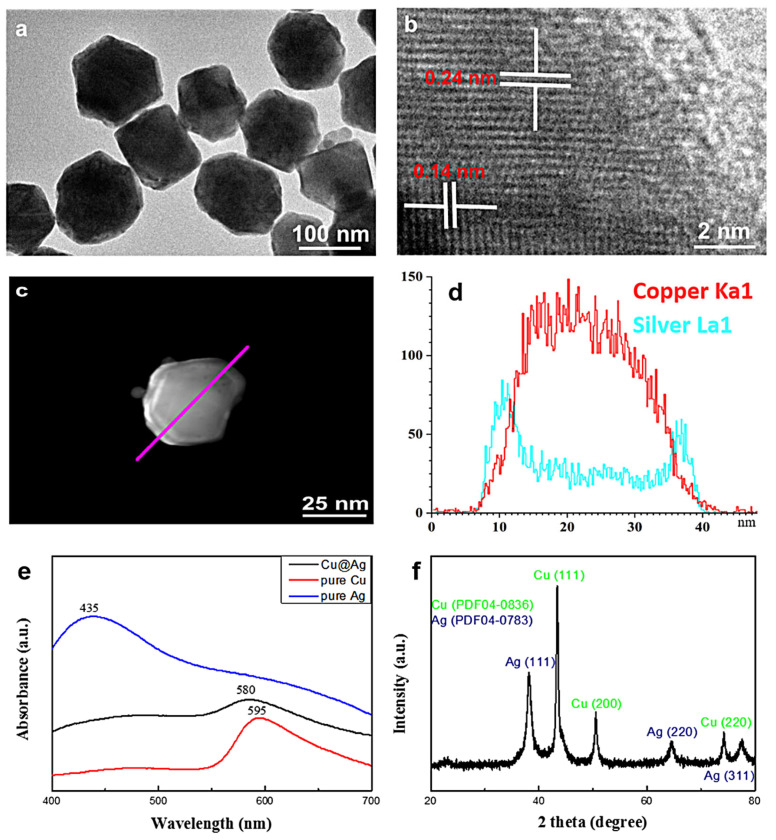
TEM images with different magnifications (**a**,**b**), HAADF-STEM image (**c**), copper and silver elemental profile along the particle diameter according to EDS analysis (**d**), UV-visible absorption spectra of the particles (**e**), and the XRD pattern of the dry powder (**f**).

Various characterization methods have been employed to investigate the nanoparticle structure. Figure 2a,b show the high-resolution transmission electron microscopy images of the Cu@Ag particles. In addition, the TEM-based SAED image proves the composition of the Cu@Ag nanoparticles (Figure S1). The particle surface appeared relatively indistinct, indicating that organic species were adsorbed on the particle surface. The magnified image revealed lattice distances of 0.18 nm in the core area and 0.24 nm near the shell area, corresponding well to the lattice spacings of Cu (220) and Ag (220) planes, respectively. Furthermore, the composition distribution of the two metal elements was confirmed by EDS analysis in scanning transmission electron microscopy (STEM) mode by scanning an individual Cu@Ag particle along its diameter (Figure 2c,d). A typical silver profile showed slightly higher intensity at the edges than at the center. Conversely, the same scan for copper showed a complementary profile with higher intensity at the center than at the edges. The average intensity of copper is higher than that of silver. Due to the effect of metal particle size, surrounding medium, and elemental composition, the surface plasmon resonance caused by the collective oscillation of surface electrons was extremely distinctive [31]. The UV-visible absorption spectra of the particles dispersed in ethanol were recorded to discuss the particle structures. The absorption spectra of the pure Cu and pure silver synthesized through the same route were demonstrated by the peaks at 595 nm and 435 nm, respectively, which agree well with the literature report [32]. However, for the Cu@Ag nanoparticles, only one plasmon peak located at 580 nm was observed, indicating the absence of individual copper and silver in the sample (Figure 2e). Furthermore, the XRD pattern of the dry powder indicated the presence of both copper and silver with a face-centered cubic (fcc) crystal structure instead of their alloy. As Figure 2f shows, typical 2θ peaks at 43.47°, 50.67°, and 74.68° are assigned to the Cu (111), Cu(200), and Cu (220) planes (JCPDS No.04-0836), while peaks at 38.12°, 64.43°, and 77.47° correspond to the Ag (111), Ag(220), and Ag (311) planes (JCPDS No.04-0783). In addition, no other peaks, particularly those of Cu₂O and CuO, can be found, suggesting that the outer Ag shell well protects the Cu without significant oxidation.

**Figure 3:**
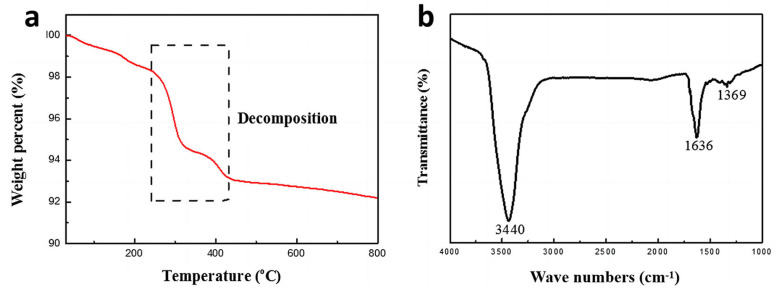
TG result of the synthesized Cu@Ag NPs under nitrogen flow (**a**), FT-IR spectra of synthesized Cu@Ag NPs (**b**).

The particle surface state was examined using thermogravimetric (TG) analysis from 30 °C to 800 °C at 10 °C/min under a nitrogen atmosphere. The number of capping layers on the Cu@AgNPs surface was estimated from the total weight loss ratio caused by the thermal decomposition of the organic residue. As shown in Figure 3a, the weight loss began at a relatively low temperature of 30 °C, indicating that the hydrophilic particles absorbed some air moisture in the washing process. The dramatic weight decrease between 220 °C and 420 °C is attributed to the loss of capping organic layers. The total weight loss of the samples was approximately 7.6%, according to the TGA results. As presented in the Fourier-transform infrared (FT-IR) spectra curve (Figure 3b), the characteristic absorption peaks of hydroxyl at 3440 cm⁻¹ and carbonyl at 1636 cm⁻¹ are observed. The hydroxyl functional group can be attributed to the byproduct of dehydroascorbic acid or the moisture absorbed from the environment [30]. As for the carbonyl group, both dehydroascorbic acid and PVP molecules contain this group. Additionally, a slight absorption peak at 1369 cm⁻¹ can be assigned to the C-N stretching group, which is strong evidence of the presence of the PVP capping layer. The FT-IR spectra demonstrate that dehydroascorbic acid and polyvinyl pyrrolidone are successfully coated on the Cu@Ag nanoparticles. The capping agent layer can effectively prevent particle aggregation and enhance oxidation resistance [30]. However, the large amount of insulative organic material may hinder interparticle connection and compromise electrical properties unless eliminated during the subsequent sintering process [33].

**Figure 4:**
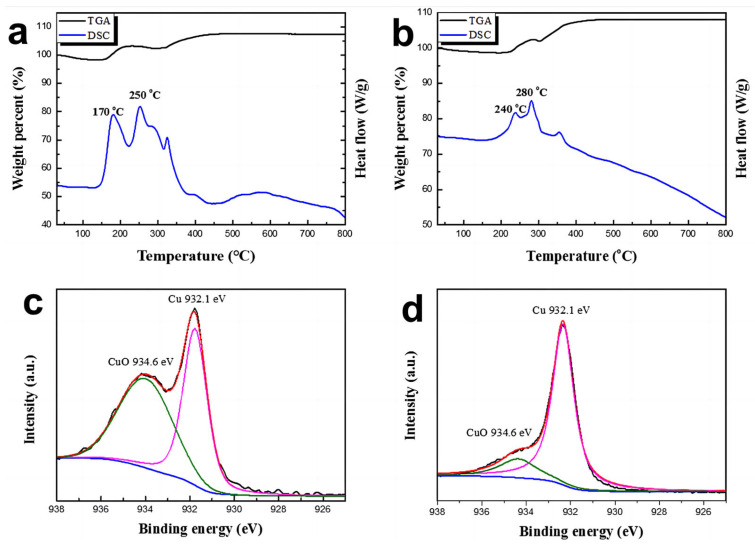
Thermal behavior of the pure Cu particle (**a**), Cu@Ag particle (**b**), XPS analysis of the Cu NPs (**c**), and the Cu@Ag NPs (**d**) both stored in ambient conditions for 60 days.

The improved oxidation resistance of Cu@Ag particles can be demonstrated by comparing the thermal behaviors of pure Cu and Cu@AgNPs in ambient conditions. As shown in Figure 4a, the pure Cu nanoparticles exhibited an initial weight increase at approximately 170 °C, which corresponds well to the exothermic peak observed in the differential scanning calorimetry (DSC) curve. This indicates that copper oxidation begins around 170 °C. The subsequent weight increase occurred at approximately 250 °C, accompanied by another exothermic peak in the DSC curves, attributed to cuprous oxide (Cu_2_O) formation. These thermal behavior results align with the reported oxidation mechanism of copper [34].

Conversely, the weight increase of Cu@Ag particles was observed at 240 °C and 280 °C, corresponding to two weak exothermic peaks in the DSC curves (Figure 4b). These peaks indicate the formation of CuO and Cu_2_O, respectively. The presence of the silver shell on Cu@Ag NPs delayed the onset of oxidation by approximately 70 °C, demonstrating significantly improved oxidation resistance. Considering that the surface oxidation layer is amorphous and small in volume, XPS analysis is suitable for probing the extent of surface oxidation of the particles. Figure 4c,d show the Cu 2p₃/₂ spectra with peak fitting of Cu and Cu@AgNPs stored in ambient conditions for 60 days, and the detail of XPS survey spectrum have shown in Figure S3. The spectrum of pure Cu exhibits two distinct peaks around 932.1 eV and 934.6 eV, corresponding to Cu and CuO, respectively. The binding energies of Cu 2p₃/₂ for pure Cu, Cu_2_O, and CuO are around 932.1 eV, 932.6 eV, and 934.6 eV, respectively [10,11,30,33]. In contrast, the peak fitting spectra of Cu@AgNPs only show one strong peak around 932.1 eV and an extremely weak peak around 934.6 eV that is almost indistinguishable. These results adequately verify that the dense Ag shell significantly enhances the oxidation resistance of Cu@AgNPs.

**Figure 5:**
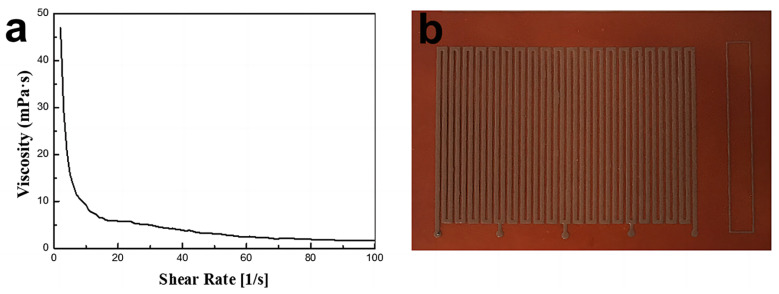
(**a**) Conductive ink viscosity at different shear rates. (**b**) Photograph of the screen print pattern on PI flexible substrate.

The Cu@AgNPs were mixed mechanically with an organic solvent of 87.5 wt% ethylene glycol and 12.5 wt% diethylene glycol. To adjust the physical/chemical properties, additives such as cellulose, propanetriol, and diethyl butyl ether were introduced into the mixture. A conductive ink with a 60 wt% loading of Cu@AgNPs was ultimately prepared. As shown in Figure 5a, the conductive ink exhibited typical non-Newtonian fluid behavior, with viscosity decreasing as the shear rate increased. The viscosity of the ink at a shear rate of 100 s⁻¹ was measured to be 1.56 mPa·s. The rheological properties indicated that this ink was suitable for silk screen printing. Using a simplified screen-printing process, a conductive circuit pattern with a 500 mm-wide line was successfully printed on the flexible PI substrate, as shown in Figure 5b.

**Figure 6:**
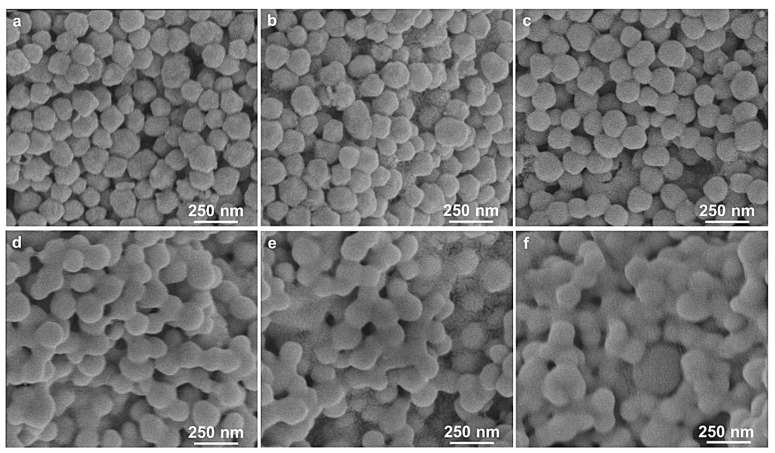
SEM images of the surface of Cu@Ag conductive ink on PI substrates with various sintering temperatures: 140 °C (**a**), 170 °C (**b**), 200 °C (**c**), 230 °C (**d**), 260 °C (**e**), and 290 °C (**f**).

To convert the intermittent nonconductive circuit into a continuous conductive one, a sintering process was necessary. In recent years, various novel and effective alternative solutions for sintering have been developed [35]. In this work, the printed pattern was sintered through conventional heat annealing for 15 minutes under a nitrogen atmosphere. Figure 6 presents the SEM images of the Cu@Ag patterns screen-printed on the PI substrate and sintered at various temperatures ranging from 140 °C to 290 °C. Evidently, when the sintering temperature was below 230 °C, the nanoparticles could only form a partially connected metal film with numerous voids. This indicates that the particle connection is solely due to van der Waals forces at low sintering temperatures, and no metallic bond is formed. As the sintering temperature was increased to 230 °C, a certain amount of necking structure, established by metal bonding, became prominently visible. With higher heat energy, the nanoparticles fused together, and adjacent particles agglomerated into small clusters, which grew coarser and denser upon annealing at 260 °C. When the sintering temperature reached 290 °C, all the particles were fully fused, resulting in ambiguous aggregate boundaries. Similar to the surface properties of bulk metal, the void count within the film significantly decreased, indicating that at 290 °C, the heat energy was sufficient to fuse the metal nanoparticles together.

**Figure 7:**
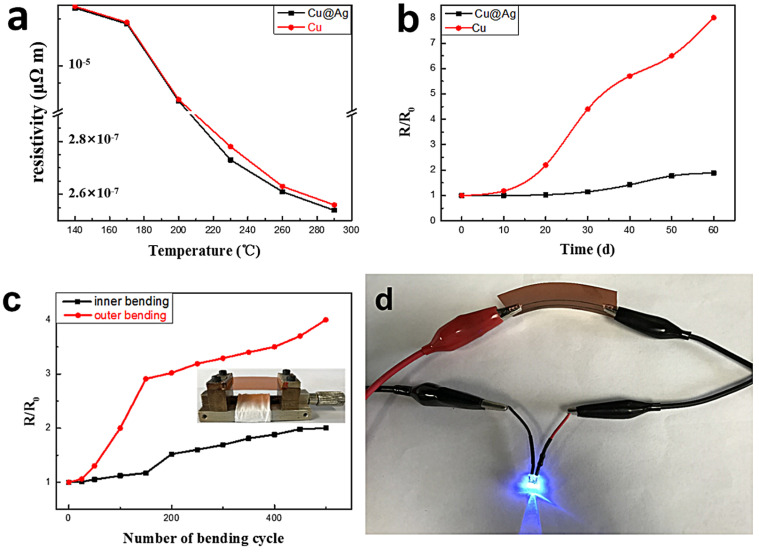
(**a**) Resistivity variations of sintering Cu and Cu@Ag films as a function of the sintering temperature. The 15 min sintering process was conducted in a rapid thermal annealing under the nitrogen atmosphere. (**b**) Relative resistance (R/R0) of the Cu and Cu@Ag patterns changes as a function of the exposure time. The patterns were exposed to ambient room conditions (approximately 25 °C 50% humidity in air atmosphere). (**c**) Relative resistance changes of the Cu@Ag pattern as a function of the bending cycle number. Inset is the image of the lab-made bending tester. (**d**) LED bulb illumination of the Cu@Ag pattern in the bending state.

The temperature-dependent resistivities of both the Cu pattern and Cu@Ag pattern were calculated and are presented in Figure 7a. The curves in Figure 7a show a sharp decrease in resistivity at 230 °C, which corresponds well with the SEM images. The resistivity of the Cu@Ag pattern decreased to 25.5 μΩ·cm, approximately 15 times the resistivity of bulk Cu, when the sintering temperature was raised to 290 °C. Compared to the resistivity of Cu@Ag patterns reported in other studies, the optimized value we obtained was slightly higher [8,27]. Regardless of the variation in sintering temperature, the resistivity of the pure Cu@Ag pattern was consistently slightly lower than that of the pure Cu pattern owing to the significantly higher diffusion rate and intrinsic bulk resistivity of Ag compared to Cu.

The environmental aging behavior of sintered Cu-based patterns, particularly their susceptibility to oxidation, poses significant challenges for practical applications. To assess the ability to maintain the original conductivity, both the pure Cu and Cu@Ag patterns were investigated. The resistivity of the patterns exposed to ambient room conditions (approximately 25 °C and 50% humidity in an air atmosphere) over time is shown in Figure 7b.

Notably, the oxidation-resistant Cu@Ag pattern exhibits a significantly different trend than the Cu pattern. After 20 days of exposure, the resistivity of the Cu pattern increased by 1.2 times, and this value continued to rise to approximately seven times after 60 days of exposure. Conversely, the resistivity of the Cu@Ag pattern only increased by 0.8 times throughout the 60-day testing period. Therefore, the aging test results demonstrate that the Ag shell can effectively protect the Cu core from moisture and oxygen, thereby mitigating oxidation and preserving the conductivity of the pattern.

To investigate the mechanical fatigue performance of the Cu@Ag pattern, bending tests were conducted using a simple experimental design. In the test, the pattern samples were fastened to two holders of the bending tester, which could move freely along a line from opposite directions while maintaining the bending radius. Figure 7c illustrates the resistance changes of the Cu@Ag pattern as a function of the bending cycle number (the inset shows the lab-made bending tester). The bending tests consisted of outer bending, which involved folding the pattern strips outside, and inner bending, which involved folding the strips inside. The relative resistance (R/R0) of the sintered Cu@Ag pattern in the two bending styles increases as the bending cycle number increases. In the inner bending test, the pattern can keep the resistivity variation relatively low when the bending cycle number is below 200. However, the critical cycle number dropped to 100 in the outer bending test. Under the same bending cycle, the increase of relative resistance of the pattern in the inner bending test is always considerably smaller than in the outer bending test. The different mechanical fatigue behavior of the Cu@Ag pattern may be caused by the distinguishing mismatch between the printed metal layer and its substrate under the two bending styles. The appearance of vast cracks is accelerated under tensile stress in outer bending but retarded under compressive stress in inner bending [36]. Previous studies show that high mechanical fatigue strength can be achieved without dramatically degrading the initial electronic property by adjusting the ink formula or pretreating the substrate surface [37,38]. To demonstrate the applicability of the Ag-coated Cu nanoparticle-based ink for printed electronic devices, a continuous coil circuit with a relatively uniform surface structure was manufactured to light up an LED. As shown in Figure 7d, the LED illumination intensity remains constant without signification attenuation, even if it is in the bending state. Thus, the present Cu@Ag ink can be used as conductive ink in printed electronics.

## 4. Conclusions

In this work, the highly monodisperse and uniformly shaped Cu@Ag nanoparticles were successfully synthesized using a novel, low-cost, large-scale, high-throughput, and environmentally friendly route. The Cu@Ag particles, protected by the inert Ag shell, exhibited excellent antioxidation performance and storage capacity. Furthermore, a well-dispersed and stable Cu@Ag-based conductive ink with suitable rheological properties was successfully screen-printed onto a flexible PI substrate, followed by an optimized sintering process. Compared to bulk copper, the electrical resistivity of the conductive pattern made by Cu@Au has declined about 15 times. Moreover, the conductive pattern demonstrated excellent bending fatigue properties. The innovative preparation route of Cu@Ag nanoparticles, combined with an optimized sintering process, will significantly enhance the applications of Cu-based conductive ink in the field of printed electronics.

## Data Availability

The data presented in this study are available from the corresponding author upon request.

## Supplementary Materials

The following supporting information can be downloaded at: https://www.mdpi.com/article/10.3390/mi14071318/s1, Figure S1: SAED pattern of Cu@Ag core-shell nanoparticles; Figure S2: Size distribution of Cu@Ag nanoparticles according to DLS; Figure S3: (**a**) survey spectra, (**b**) Ag 3d spectra, (**c**) Cu 2p_3/2_ spectra.
