# Supplementary material for: Facile Preparation of Monodisperse Cu@Ag Core–Shell Nanoparticles for Conductive Ink in Printing Electronics
Source: Micromachines (Basel). 2023 Jun 27;14(7):1318. doi: 10.3390/mi14071318 (PMC10383072; doi:10.3390/mi14071318)
Supplement: Supplementary file 1 [file micromachines-14-01318-s001.zip › micromachines-2431146-supplementary.pdf]

Supporting information for

Facile preparation of monodisperse Cu@Ag core–shell nanoparticles for conductive ink in printing electronics

Gang Li [1,2,3], Xuecheng Yu [2], Ruoyu Zhang [2], Qionglin Ouyang [2], Rong Sun [2], Liqiang Cao [1*], and Pengli Zhu [3*]

[1] System Packaging and Integration Research Center, Institute of Microelectronics of Chinese Academy of Sciences, Beijing 100029, China

[2] Shenzhen Institute of Advanced Electronic Materials, Shenzhen Institutes of Advanced Technology, Chinese Academy of Sciences, Shenzhen 518055, China

[3] University of Chinese Academy of Sciences, Beijing 100049, China

* Correspondence: caoliqiang@ime.ac.cn, pl.zhu@siat.ac.cn

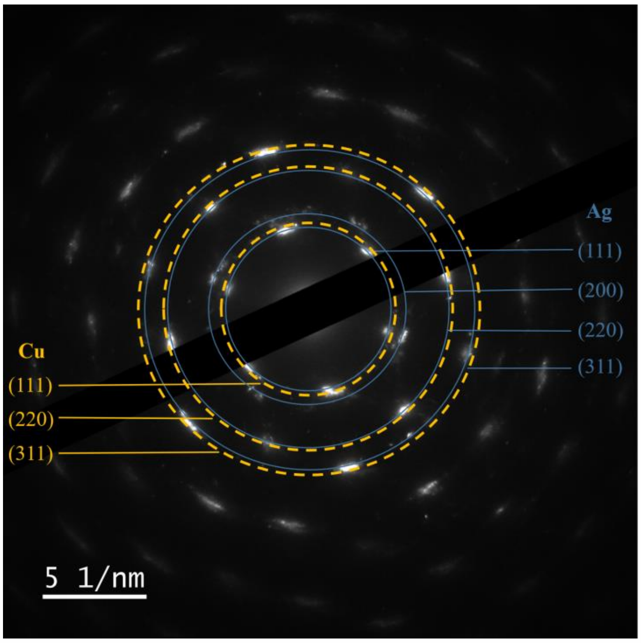

Figure S1. SAED pattern of Cu@Ag core-shell nanoparticles.

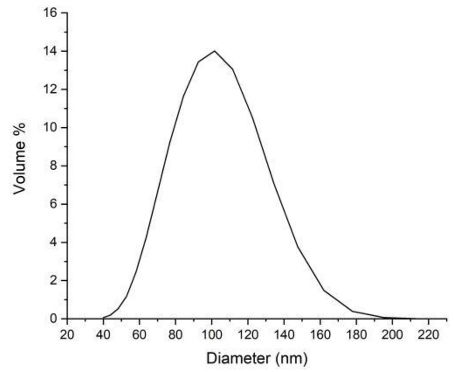

Figure S2. Size distribution of Cu@Ag nanoparticles according to DLS.

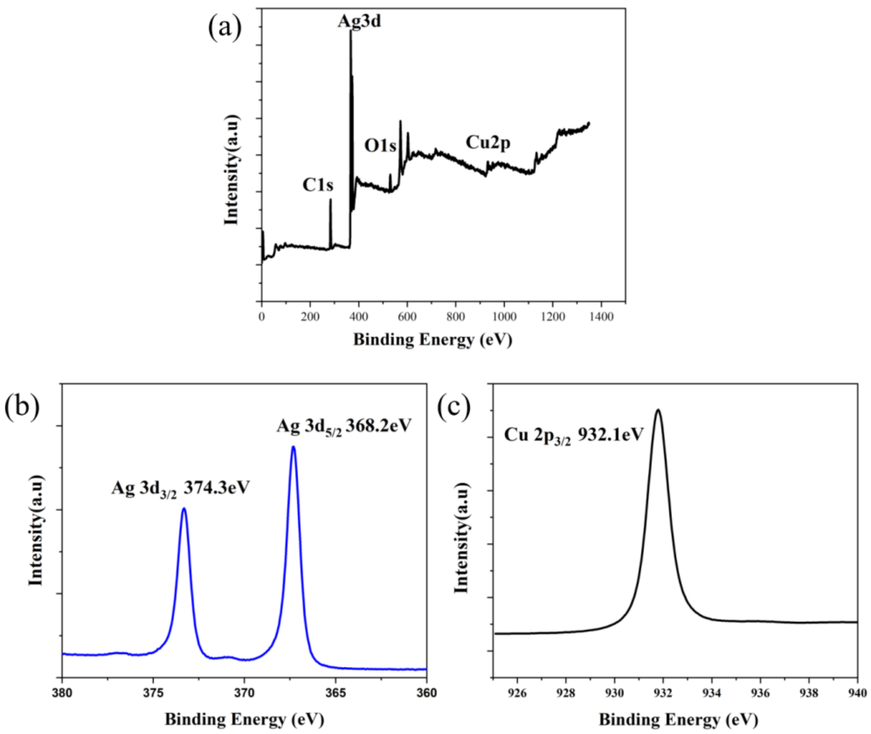

Figure S3. (**a**) survey spectra, (**b**) Ag 3d spectra, (**c**) Cu 2p3/2 spectra.

XPS analysis was further conducted to characterize the element composition of the different valence states of Cu–Ag core–shell nanoparticle. Figure S3 clearly illustrates that the binding energies of Cu 2p3/2, Ag 3d5/2 and Ag 3d3/2 were 932.1eV, 368.2eV and 374.3eV, respectively, demonstrating the presence of elementary Cu(0) and Ag(0).
